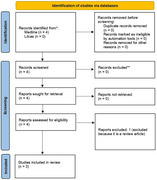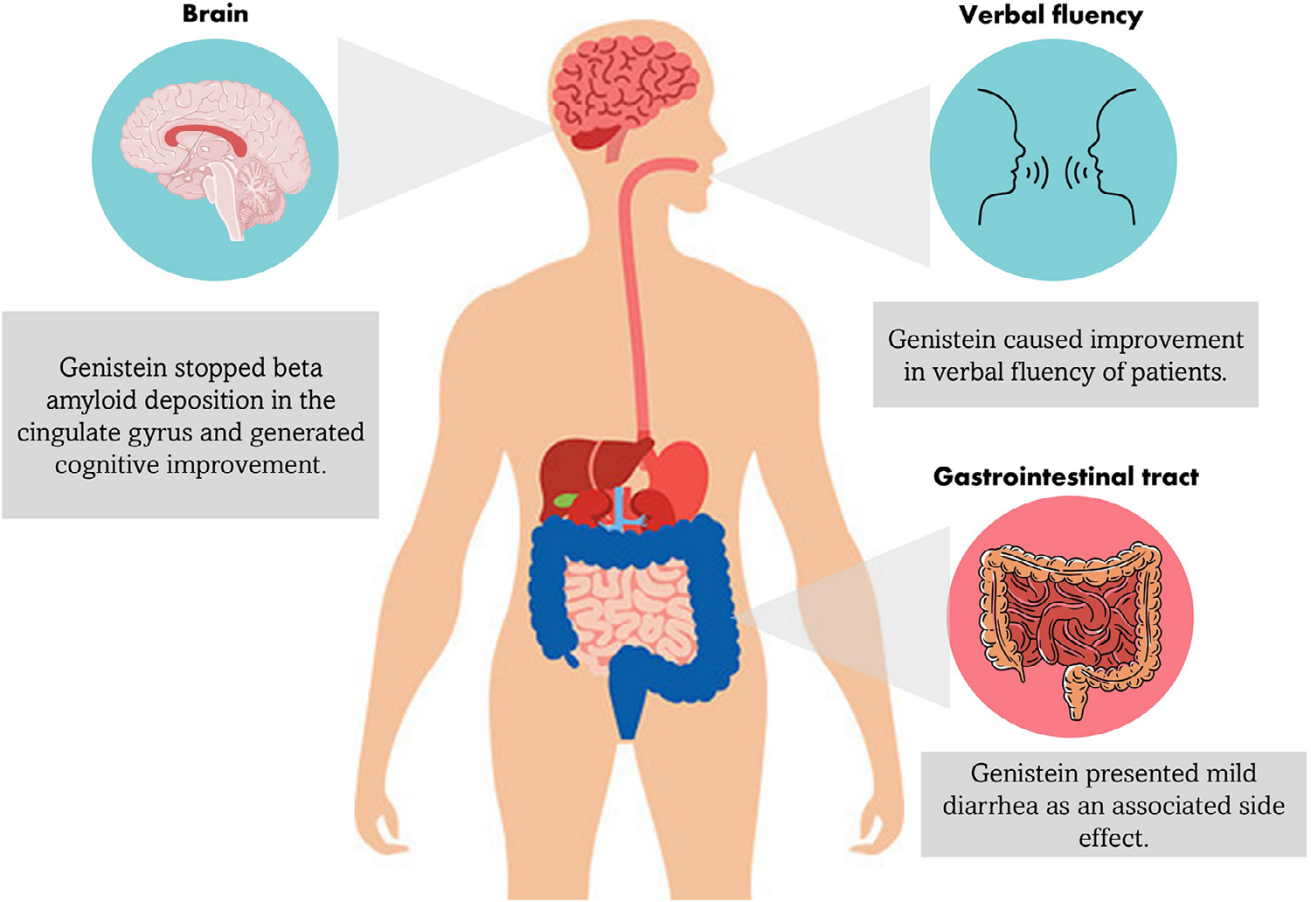# Genistein as a Therapeutic Agent in the Prodromal Phase of Alzheimer's Disease: Systematic Review

**DOI:** 10.1002/alz70859_103969

**Published:** 2025-12-26

**Authors:** Kevin Gustavo dos Santos Silva, Vinícius Ferreira Souza, Grazielle Larissa Fonte Alves, Carlos Rocha Oliveira

**Affiliations:** ^1^ Anhembi Morumbi University, São José dos Campos, São Paulo Brazil; ^2^ Federal University of São Paulo, São José dos Campos, São Paulo Brazil

## Abstract

**Background:**

Alzheimer’s disease, the most prevalent cause of dementia globally, imposes a tremendous public health burden, with cases projected to triple by 2050. Among potential therapeutic avenues, genistein ‐ a phytoestrogen with antioxidant and anti‐inflammatory Properties ‐ has garnered attention for its role in modulating amyloidβ deposition and promoting neuronal survival. This systematic review aims to evaluate its efficacy in the prodromal phase of Alzheimer's disease, a critical window for intervention.

**Method:**

This study is a systematic review of randomized clinical trials on the effect of genistein in the prodromal phase of Alzheimer's disease. The search strategy involved an active search in the Medline and Lilacs databases, using the term "Genistein" associated with "Alzheimer's disease" by the Boolean operator "AND." Filters were applied for randomized clinical trials published in English and Spanish, with articles dated up to 15 years ago. Trials were included if they evaluated genistein in participants diagnosed with prodromal Alzheimer's disease according to standardized criteria (e.g., Dubois). The identified articles were assessed by two independent reviewers. Risk of bias was assessed using the Revised Cochrane Risk‐of‐Bias Tool for Randomized Trials (RoB 2.0).

**Result:**

In total, three (3) clinical trials were evaluated (Figure 1). The study by Gleason et al. (2015) revealed an association between plasma levels of isoflavones and speed, dexterity and verbal fluency (Figure 2). The analysis of beta‐amyloid deposition carried out in the study by Viña et al. (2022) showed that patients treated with genistein did not increase their uptake in the anterior cingulate gyrus after treatment (*p* = 0.878), while those treated with placebo increased it (*p* = 0.036). Finally, the study by Wang et al (2020) shows that Genistein preserved cognitive function in verbal learning and executive function.

**Conclusion:**

Genistein has been shown to improve cognitive function and reduce beta‐amyloid deposition in patients with prodromal Alzheimer's disease. However, small sample sizes and variability in dosages limit the generalizability of these findings. Future trials with larger cohorts and standardized protocols are needed to validate these results.